# Intraoperative monitoring of cerebrovascular autoregulation in infants and toddlers receiving major elective surgery to determine the individually optimal blood pressure – a pilot study

**DOI:** 10.3389/fped.2023.1110453

**Published:** 2023-02-14

**Authors:** Maximilian Iller, Felix Neunhoeffer, Lukas Heimann, Julian Zipfel, Martin U. Schuhmann, Simon Scherer, Markus Dietzel, Joerg Fuchs, Michael Hofbeck, Stefanie Hieber, Frank Fideler

**Affiliations:** ^1^ Department of Anesthesiology and Intensive Care Medicine, Pediatric Anesthesiology, University Hospital Tuebingen, Tuebingen, Germany; ^2^ Department of Pediatric Cardiology, Pulmonology and Pediatric Intensive Care Medicine, University Children’s Hospital Tuebingen, Tuebingen, Germany; ^3^ Department for Internal Medicine, Hospital Herrenberg, Herrenberg, Germany; ^4^ Section of Pediatric Neurosurgery, Department of Neurosurgery, University Hospital Tuebingen, Tuebingen, Germany; ^5^ Department of Pediatric Surgery and Pediatric Urology, University Children’s Hospital Tuebingen, Tuebingen, Germany

**Keywords:** cerebrovascular autoregulation (CAR), optima mean arterial blood pressure (MAPopt), blood pressure limits of autoregulation (LAR), near-infrared spectroscopy (NIRS), regional cerebral oxygen saturation (rSO2), local relative total hemoglobin levels (rTHb), hemoglobin volume index (HVx), pediatric general anesthesia

## Abstract

**Introduction:**

Inducing general anesthesia (GA) in children can considerably affect blood pressure, and the rate of severe critical events owing to this remains high. Cerebrovascular autoregulation (CAR) protects the brain against blood-flow-related injury. Impaired CAR may contribute to the risk of cerebral hypoxic–ischemic or hyperemic injury. However, blood pressure limits of autoregulation (LAR) in infants and children are unclear.

**Materials and methods:**

In this pilot study CAR was monitored prospectively in 20 patients aged <4 years receiving elective surgery under GA. Cardiac- or neurosurgical procedures were excluded. The possibility of calculating the CAR index hemoglobin volume index (HVx), by correlating near-infrared-spectroscopy (NIRS)-derived relative cerebral tissue hemoglobin and invasive mean arterial blood pressure (MAP) was determined. Optimal MAP (MAPopt), LAR, and the proportion of time with a MAP outside LAR were determined.

**Results:**

The mean patient age was 14 ± 10 months. MAPopt could be determined in 19 of 20 patients, with an average of 62 ± 12 mmHg. The required time for a first MAPopt depended on the extent of spontaneous MAP fluctuations. The actual MAP was outside the LAR in 30% ± 24% of the measuring time. MAPopt significantly differed among patients with similar demographics. The CAR range averaged 19 ± 6 mmHg. Using weight-adjusted blood pressure recommendations or regional cerebral tissue saturation, only a fraction of the phases with inadequate MAP could be identified.

**Conclusion:**

Non-invasive CAR monitoring using NIRS-derived HVx in infants, toddlers, and children receiving elective surgery under GA was reliable and provided robust data in this pilot study. Using a CAR-driven approach, individual MAPopt could be determined intraoperatively. The intensity of blood pressure fluctuations influences the initial measuring time. MAPopt may differ considerably from recommendations in the literature, and the MAP range within LAR in children may be smaller than that in adults. The necessity of manual artifact elimination represents a limitation. Larger prospective and multicenter cohort studies are necessary to confirm the feasibility of CAR-driven MAP management in children receiving major surgery under GA and to enable an interventional trial design using MAPopt as a target.

## Introduction

Severe critical events occur in approximately >5% of children receiving general anesthesia (GA) (1, 2), hypotension with the risk of impaired cerebral perfusion accounts for a relevant proportion.

Avoiding inadequate mean arterial blood pressure (MAP) values could contribute to the reduction of perioperative neurological complications because MAP is the driving force for an adequate cerebral perfusion (3, 4). Several prospective observational studies in children have investigated the effect of intraoperative MAP changes on cerebral oxygen saturation measured by near-infrared spectroscopy (NIRS) and on cerebral blood flow (CBF). As MAP falls below certain levels, the risk of a decrease in CBF and subsequent hypoperfusion leading to transient or permanent neuronal damage increases (3, 4).

Cerebrovascular autoregulation (CAR) is mediated by cerebral precapillary vasoconstriction or vasodilation occurring in response to changes in slow waves in MAP and aims to ensure a constant CBF over a certain MAP range within the limits of autoregulation (LAR) and protects the brain against injury from hypo- or hyperperfusion. Thus, the concept of CAR is defined by lower limit of autoregulation (LLA) and upper limit of autoregulation (ULA). CAR impairment due to morbid processes or the occurrence of MAP values below or above the LLA or ULA, respectively, can lead to both hypo- and hyperperfusion that increases the risks of brain injury and transient or persistent neurological disability (5, 6).

Maintaining MAP at an optimal point within the LAR leads to the concept of MAPopt, which is the MAP with the highest and best preserved pressure reactivity and thus, the best functionality of CAR to enable optimal CBF (7, 8). Postoperative retrospective studies showed better neurologic outcome if MAP was closer to MAPopt (9).

However, generally applicable MAP values to maintain MAP within LAR in infants, toddlers, and children are unknown and may not even be applicable to all children of a specific age and weight.

NIRS is a non-invasive, continuous, and accessible monitoring tool that allows direct measurement of regional cerebral oxygen saturation (rSO_2_) and local relative total hemoglobin level (rtHb) (10, 11). Autoregulatory vasodilation and vasoconstriction result in changes in cerebral blood volume, which are associated with changes in the rtHb. The correlation of rtHb levels and MAP leads to the hemoglobin volume index (HVx), a noninvasively determined and well established surrogate parameter for cerebrovascular reactivity (12, 13).

According to Lee et al., non-invasive cerebrovascular autoregulation monitoring with HVx is an excellent alternative to the best established and evaluated CAR index PRx, which is based on invasively measured intracranial pressure (ICP) as a surrogate parameter for cerebral blood volume. Slow changes in the rtHb measured by NIRS are caused by the same blood volume changes that cause slow waves of ICP used for calculating the ICP-derived index PRx (14).

CAR monitoring has been validated in animal models and in adult and pediatric trials (5, 14–19).

In a pediatric swine model, the NIRS-derived index HVx accurately detected the lower limit of autoregulation (LLA) compared to laser-Doppler flow measurements (20).

Impaired CAR results in higher index values, whereas intact CAR is indicated by HVx values approaching zero or even becoming negative (12, 17, 18).

The aims of this pilot study were to describe the feasibility of CAR monitoring using HVx during major elective surgery in toddlers and small children under GA to determine MAPopt and lower and upper blood pressure limits of autoregulation as a guide for blood pressure management.

## Materials and methods

In total, 20 consecutive infants, toddlers, and small children receiving major elective surgery were prospectively enrolled from January 2019 to April 2020. The inclusion criteria were age <4 years and received major elective pediatric surgery requiring GA. Meanwhile, the exclusion criteria were cardiac or neurosurgical interventions. As no intervention was performed, the study was not registered in an international research platform. Data were prospectively collected with institutional review board approval (763/2016BO1), and the study was conducted in compliance with the Declaration of Helsinki. Written parental consent for all the patients was obtained in advance.

Demographic data comprised age at surgery, preoperative weight and height, sex, diagnosis, and surgical procedure. Autoregulation monitoring was initiated as part of routine monitoring. Adverse events were documented by the anesthetist in charge.

Anesthesia was performed by experienced pediatric anesthesiologists. Two times as total intravenous anesthesia using continuous infusion of propofol and remifentanil, eighteen times as balanced anesthesia using a combination of sevoflurane and continuous infusion of remifentanil.

All the patients were subjected to a standardized pediatric anesthetic treatment protocol. Hemodynamic therapy was actively managed using invasive arterial blood pressure, SPO_2_ monitoring, electrocardiogram, central venous saturation, serum lactate, and diuresis monitoring. Additionally, perfusion status was monitored by standardized capillary refill time. Therapeutic goals were MAP > 40 mmHg in infants <6 months, MAP > 50 mmHg in infants 6–12 months, and MAP > 55 mmHg in toddlers and children aged 1–4 years. Additional therapeutic targets included a difference between arterial oxygen saturation and central venous saturation of 30%, diuresis >2 ml/kg/h, capillary refill time <2 s, and lactate <2 mmol/L.

The attending anesthetists were blinded to the calculations of MAPopt, LLA, and ULA. Thus, CAR monitoring did not have any influence on procedures.

For cerebral monitoring, a two-channel INVOS™ 510°C cerebral/somatic oximeter (Medtronic) was used. OxyAlert™ NIRSensors, with a two-wavelength LED source (730 and 810 nm) and two photodiode detectors with source-detector separations of 30 and 40 mm, were used for measuring rSO_2_ and relative total hemoglobin (rtHb) with a sensor placed on the forehead, lateral to the midline. Monitoring was performed continuously during GA. The invasive blood pressure monitor and the cerebral oximeter were connected to a laptop running the ICM + software (Cambridge Enterprises, Cambridge, UK) for data recording at 100 Hz as described previously (17). Artifacts in the NIRS (e.g., accidental detachment of the sensor) and MAP (e.g., blood gas sampling and calibration/arterial line flushes) signals were removed manually prior to data analysis (a period of 1 min prior and 5 min past the artifact were excluded from data evaluation). Electrocautery did not influence the measurement. Since HVx was determined based on invasively measured MAP, a plausibility check *via* non-invasive blood pressure was performed repetitively, and a deviation of >5 mmHg resulted in the re-calibration of the MAP measurement. NIRS data were recorded continuously. Basic monitoring data, including blood gas analysis and ventilation, were recorded initially and at least every 30–60 min.

**Figure 1:**
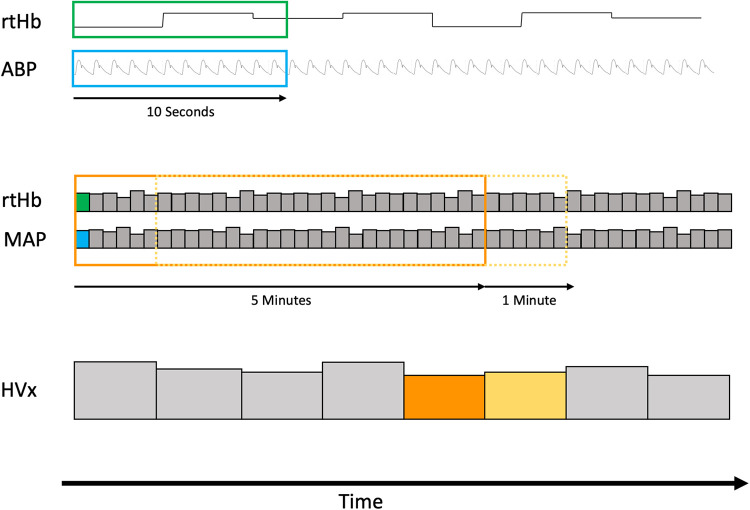
Schematic diagram of how the hemoglobin volume index (HVx) is calculated. The first row demonstrates raw input data of continuous arterial blood pressure and relative total hemoglobin (rtHb) at their respective sampling frequency. Raw data are averaged over 10 s. In total, 30 pairs or 5 min of MAP and rtHb were used to calculate the first HVx. For each minute, a successive HVx is than calculated including the last 30 pairs.

HVx was calculated with a continuous moving Pearson correlation between MAP and rtHb (14, 21). rtHb is inversely proportional to the transmittance of light with a wavelength of 805–810 nm, which is isosbestic to both oxy- and deoxyhemoglobin, an optical density that is reported in the RS232 data stream from the INVOS monitor (rtHb = 1 − optical density A * 50). rtHb is not affected by fluctuations in oxygen saturation. In the HVx, autoregulatory vasoconstriction and vasodilation may lead to changes in cerebral blood volume that are proportional to changes in relative tissue hemoglobin. Consecutively paired 10-s averaged values of 5-min duration were used for index calculation (Figure 1). HVx is a continuous variable that ranges from −1 to +1. Optimal MAP was generated from a HVx/MAP plot as previously described (22, 23). Index values from the complete procedure time were sorted into 5 mmHg size groups of MAP, and bar graphs were generated. MAPopt was calculated as the group with the most negative HVx value (nadir of a *U*-shaped curve). The LLA was identified as the group with HVx ≥ 0.3 when the bar graph demonstrated increasing index values as MAP decreased. The ULA was defined as the group with HVx ≥ 0.3 when the bar graph demonstrated increasing index values as MAP increased (14, 21, 24). The generated HVx/MAP-curve demonstrated a *U*-shape and allowed determining MAPopt; the LLA and ULA depend on spontaneous blood pressure fluctuations of the patient over a wider MAP range, as blood pressures with both intact and impaired CAR must be covered for the algorithm to determine the above mentioned values.

SigmaPlot 2000 (SPSS, Chicago, IL, United States) was used to analyze data and create all charts. Continuous data are presented as mean (±SD), whereas categorical data are presented as percentages. Distribution was tested using an analysis of variance (ANOVA) and Shapiro–Wilk test, including a subgroup analysis. Further, multiple pairwise comparison methods including Dunn's and Holm–Sidak methods were used. *T*-test and Mann–Whitney rank sum test were used to determine statistical significance, which was set at *p* < 0.05.

## Results

Altogether, 20 patients were enrolled, of whom nine were male and eleven were female. The mean patient age and weight were 14 ± 10 months and 9 ± 3 kg, respectively.

Preoperatively, 50% of the patients were classified as American Society of Anesthesiology (ASA) 3, 40% as ASA 2, and 10% as ASA 1. The cumulative recording time was 62 h with a mean duration of operation of 186 ± 75 min. Five procedures were endoscopically performed, whereas the others were performed conventionally. None of the recordings had to be stopped or interrupted for technical issues with the sensors or arterial catheter.

MAPopt could be determined in 19 of 20 patients (95%). As some patients had not experienced the entire autoregulated blood pressure range, either LLA or ULA could not be determined. LLA could not be identified in 15% and ULA in 20% of the patients. All the obtained autoregulatory parameters are listed in Table 1. The mean MAPopt was 62 ± 12 mmHg, and the mean LLA and ULA were 55 ± 9 mmHg and 72 ± 11 mmHg, respectively. MAPopt can significantly differ to some extent even in patients with similar weight and age; the MAPopt of one patient was equivalent to LLA in another patient despite similar demographics. The complete blood pressure range with intact autoregulation could be determined in 11 of 20 patients (55%) and averaged a span of 19 ± 6 mmHg (Table 2).

**Table 1:** Patient demographics.

Patient	Weight [kg]	Age [months]	OP-time [min]	Diagnosis	Sex
1	8.5	6	262	CPAM	F
2	7.1	6	236	CPAM	F
3	9.1	21	294	Neuroblastoma	F
4	3.3	0	117	Diaphragmatic hernia	F
5	15.0	39	85	Wilms tumor	F
6	6.3	11	93	s.p. NEC	F
7	10.0	19	174	Neuroblastoma	F
8	13.6	27	233	M. Hirschsprung	M
9	3.4	0	65	Omphalocele	M
10	7.3	9	229	Hepatoblastoma	F
11	6.7	7	192	CPAM	M
12	6.8	4	192	Anal atresia	M
13	11.0	10	200	Neuroblastoma	F
14	10.0	29	177	Rhabdomyosarcoma	M
15	4.1	5	52	s.p. NEC	M
16	12.0	18	131	Neuroblastoma	F
17	11.8	24	239	Neuroblastoma	M
18	7.8	10	180	CPAM	F
19	9.0	12	271	Neuroblastoma	M
20	13.0	22	293	Portal vein thrombosis	M
Mean	8.8	14	185.8		Count M: 9
SD	3.3	11	74.4		Count F: 11

The first column is the patient number which matches with the numbers in the second table. The units of the columns are indicated in [ ]. CPAM, congenital pulmonary airway malformation. NEC, necrotizing enterocolitis.

**Table 2:** Identified characteristics of cerebral blood flow autoregulation in each patient.

Patient	MAP_opt_ [mmHg]	LLA [mmHg]	ULA [mmHg]	Range [mmHg]	Age [months]
4	50		65		0
9	39		60		0
12	52	40			4
15					5
1	67	55			6
2	62		75		6
11	73	50			7
10	60	50	70	20	9
13	57	50	65	15	10
18	76	65			10
6	56				11
19	62	50	75	25	12
16	72	60	80	20	18
7	56	50	65	15	19
3	61	55	70	15	21
20	66	50	80	30	22
17	73	60	85	25	24
8	48	40	55	15	27
14	59	55	65	10	29
5	89	75	95	20	39
Mean	62	55	72	19	14
SD	12	9	11	6	11

The patients are sorted according to their age in months. The gray fields indicate unobtainable values. ULA, upper limit of autoregulation; LLA, lower limit of autoregulation. The range is the difference between ULA and LLA. The units are indicated in [ ].

**Figure 2:**
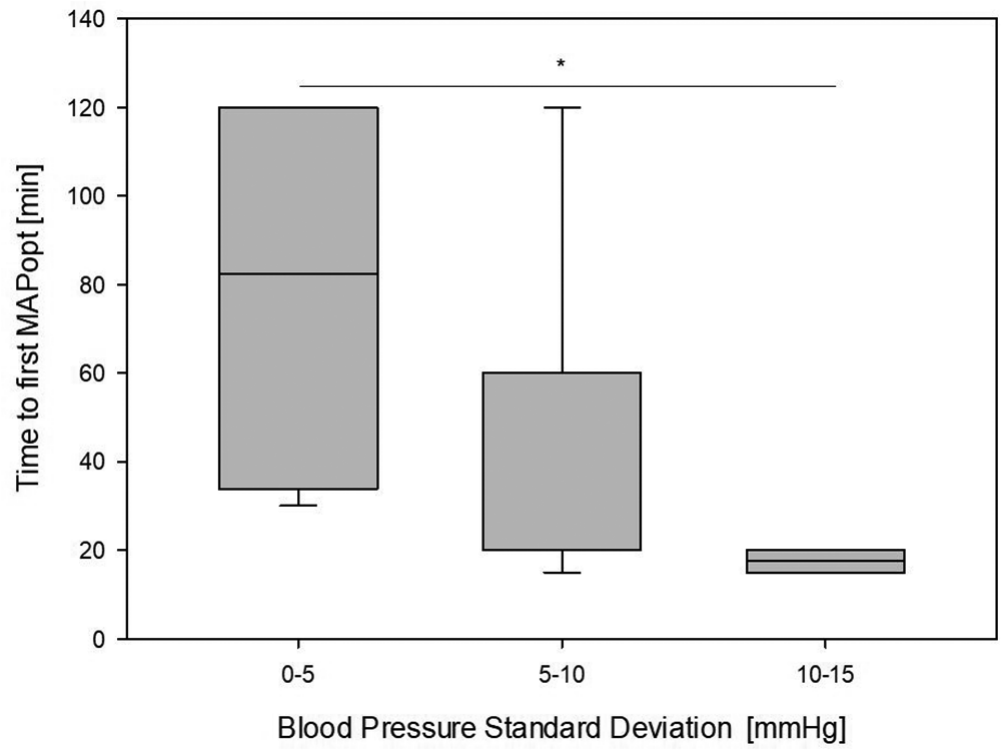
Time required to initially identify the optimal mean arterial blood pressure (MAPopt) on the *y*-axis. On the *x*-axis, the patients are grouped according to their extent of spontaneous blood pressure fluctuations depicted as blood pressure standard deviation. In patients with higher fluctuations the initial value could be identified quicker. According to the Shapiro–Wilk test, the data are normally distributed. The analysis of variance indicates statistical significance (*p* = 0.014). The bar with * indicates significant results in the subgroup analysis.

**Figure 3:**
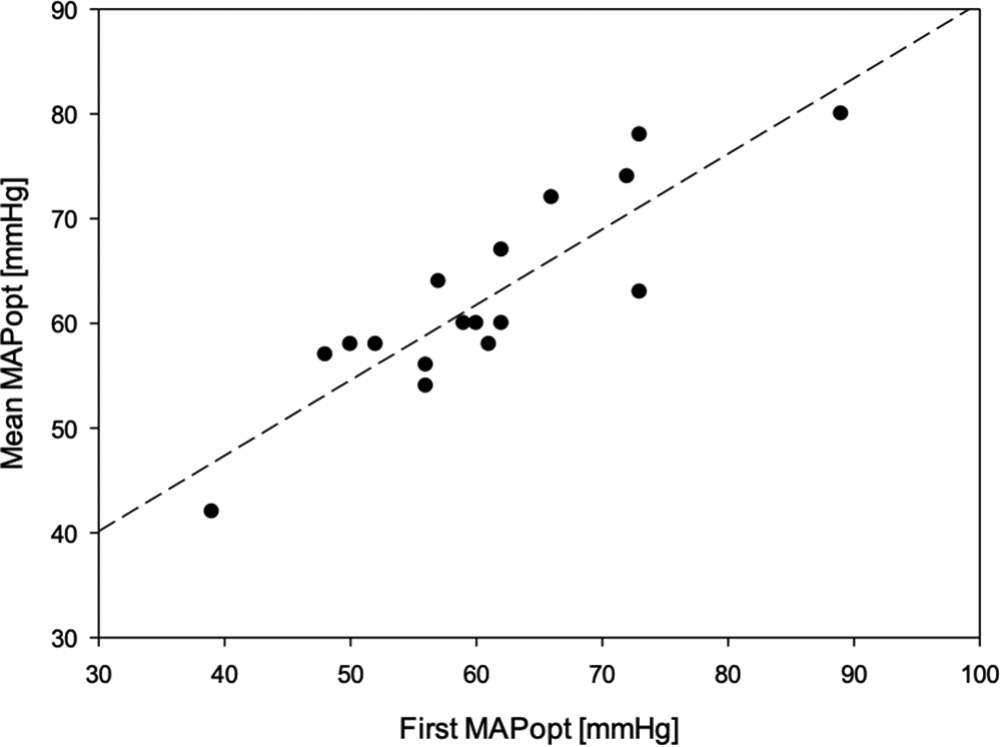
Scatterplot of the optimal mean arterial blood pressure (MAPopt), which could be identified throughout the complete surgery on the *y*-axis and the initially identified MAPopt on the *x*-axis. A linear regression analysis was performed, and a dotted regression line was added to the graph. The correlation coefficient is *R* = 0.881.

The time until a first reliable MAPopt could be identified showed significant variation. Further analyses revealed an inverse correlation between spontaneous fluctuations in blood pressure, which were operationalized as blood pressure standard deviation and the time required for a first optimal blood pressure. Hence, if patients had volatile hemodynamics, a first value was obtained quicker than in stable patients. For six patients with a blood pressure standard deviation of >10 mmHg, a first reliable MAPopt could be determined after 18 ± 3 min. By contrast, five patients with a blood pressure standard deviation of <5 mmHg required 79 ± 48 min of recording before a first MAPopt could be identified, which was significantly different (*p* = 0.014) according to the Kruskal–Wallis analysis. Figure 2 presents a boxplot of the time required for an initial MAPopt depending on those spontaneous blood pressure fluctuations. The initial MAPopt absolutely deviated 5 ± 3 mmHg from the MAPopt throughout the procedure. Figure 3 presents a scatterplot of the first and mean MAPopt.

**Figure 4:**
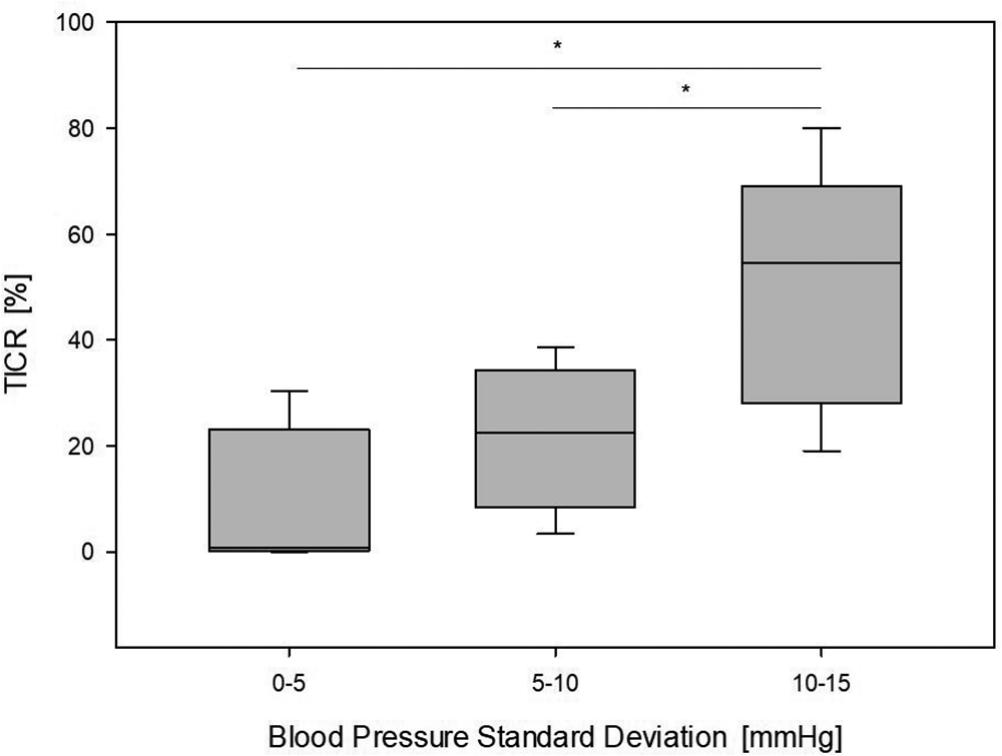
Percentage of time that the actual patient blood pressure was outside the identified optimal range which is called time in critical region (TICR%). On the *x*-axis, the patients are grouped according to the extent of their spontaneous blood pressure fluctuations depicted as blood pressure standard deviation. The patients with higher fluctuations spent a higher proportion of time outside the autoregulated range. According to the Shapiro–Wilk test and Brown–Forsythe test, the data are normally distributed. The analysis of variance indicates statistical significance of data (*p* = 0.004). The bar with * indicates significant results in the subgroup analysis.

On average, MAP was outside the range of functional CAR (HVx > 0.3) in 30% ± 24% of the operation time, denoted as “time in critical region” (TICR, Figure 4). The TICR MAP was below the LLA in 65% and above the ULA in 35%. Regarding the time outside functional CAR, a difference according to the blood pressure standard deviation was observed (Figure 4). Patients with a small (0–5 mmHg) and broad (10–15 mmHg) standard deviation had a TICR of 11% ± 17% and 51% ± 22%, respectively, which was significantly different (*p* = 0.004).

**Figure 5:**
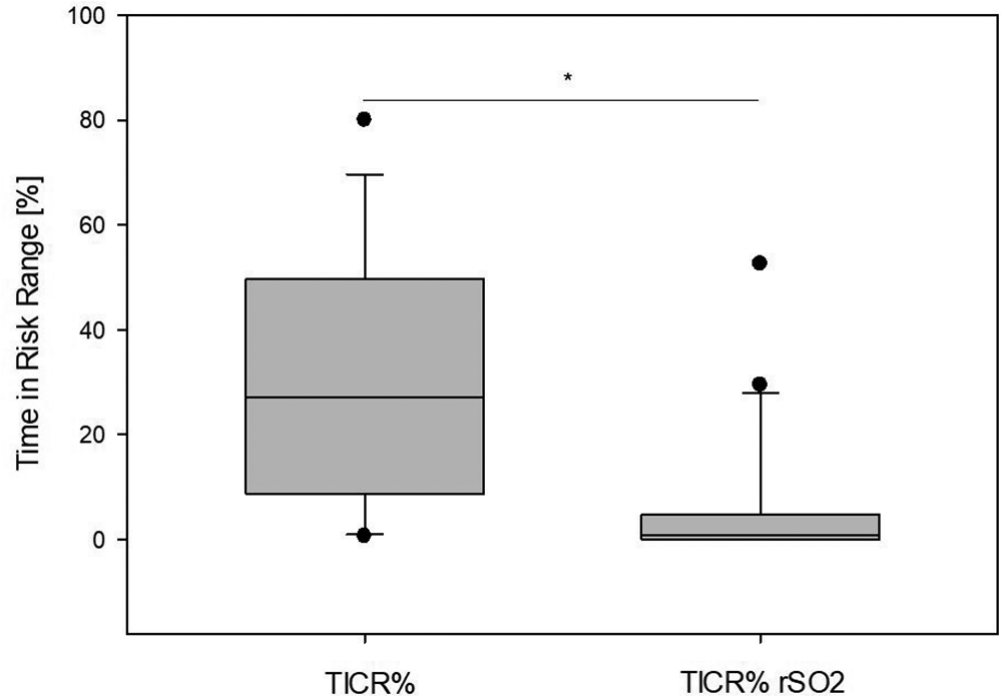
Two means of cerebral perfusion monitoring were compared. The left box indicates the percentage time of actual blood pressure outside the identified autoregulated range (TICR%). The right box indicates the percentage time when cerebral oximetry decreased by >20% from the baseline value (TICR% rSO_2_). According to the Shapiro–Wilk test, the data are non-normally distributed. Therefore, the Mann–Whitney rank sum test was performed, which revealed significant differences in mean values (*p* < 0.001).

Intraoperative cerebral oximetry monitoring is a standard method for identifying possible critical cerebral hypoxia during pediatric surgery. A decrease in cerebral rSO_2_ of >20% from baseline is considered critical (25). According to rSO_2_ monitoring, a >20% decrease from the baseline occurred in 6% ± 13% of the time compared to a TICR of 30% ± 24% according to HVx monitoring. Figure 5 presents a boxplot comparing TICR derived from cerebral oximetry and autoregulation monitoring. The Mann–Whitney rank-sum test revealed significant differences between the two modalities (*p* < 0.001). Moreover, the rSO_2_ TICR value includes a potential outlier who expended 53% of the procedure time and 20% below the baseline range, although it appeared totally stable according to cerebral autoregulation monitoring with only 0.6% TICR.

**Figure 6:**
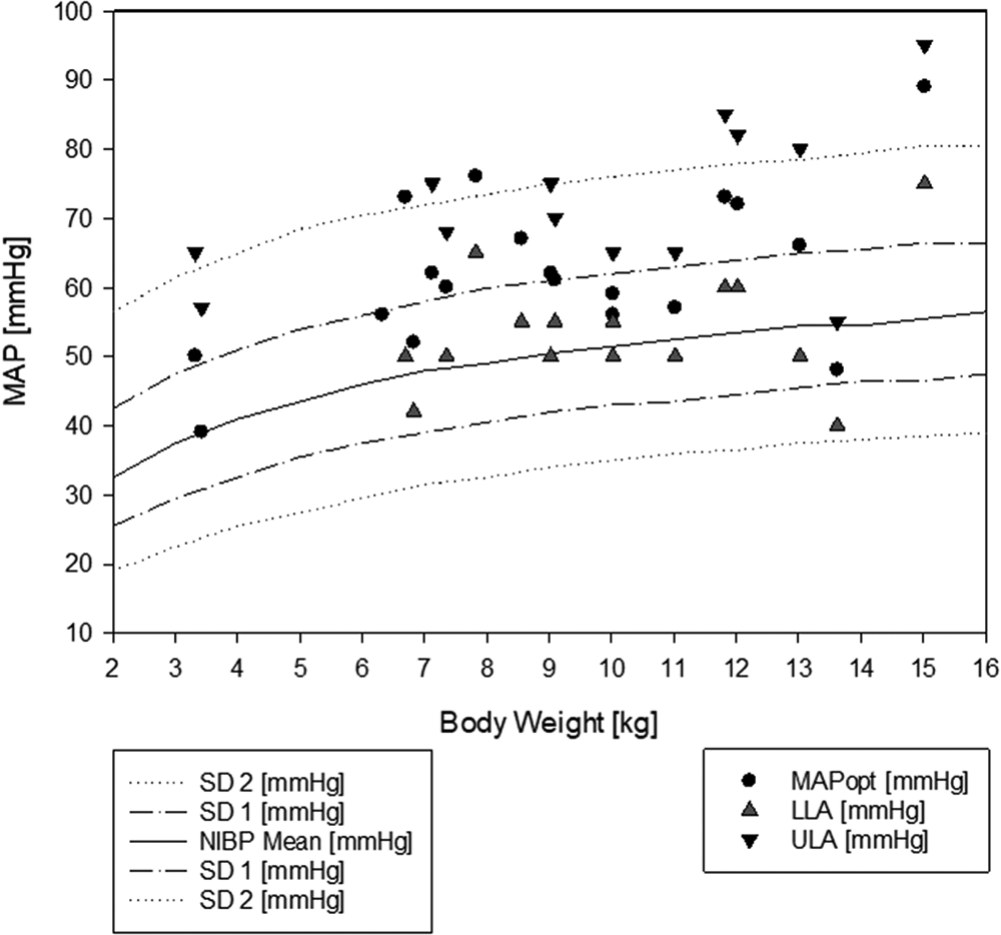
Weight-adjusted blood pressure recommendations from the literature with the identified autoregulated blood pressure range. In the background, weight-adjusted mean blood pressure recommendations from de Graaff (26) are drawn as lines of the mean and first and second standard deviation of the mean blood pressure. In the foreground, the autoregulation parameters are plotted according to the actual patient weight. MAPopt, optimal mean arterial blood pressure; LLA, lower limit of autoregulation; ULA, upper limit of autoregulation.

The identified MAPopt values were also compared to weight adjusted recommendations (26) (Figure 6). The determined MAPopt was 12 ± 8 mmHg higher than the values recommended by de Graaff. In 10 out of 15 patients in whom LLA could be determined, LLA was above the recommended value from the literature.

## Discussion

As inducing GA in children can considerably affect blood pressure (27–30), and the rate of severe critical events in children receiving anesthesia remains high (1, 2), monitoring techniques that serve as a guide for hemodynamic management to improve safety and reduce neurodevelopmental morbidity are urgently needed.

A functional CAR mechanism protects the brain against low flow or ischemic injury resulting from transient fluctuations in arterial blood pressure within the LAR (27), whereas an impaired CAR or MAPs outside the limits of CAR increase the risk of hypoxic–ischemic or hyperemic brain injury (27, 31).

We performed CAR monitoring in 20 infants, toddlers, and small children receiving major surgery under GA and demonstrated that non-invasive CAR monitoring, using the NIRS-derived HVx provides robust data and can be performed reliably and safely in clinical practice.

The key observations in this study are as follows. First, MAP was outside the range of intact autoregulation (TICR) for 30% of the monitoring time. Second, MAPopt could be determined in 95% of the patients. Third, MAP was more often below than above MAPopt. Finally, in ≥50% of the patients, the LLA was higher than the age-adjusted guideline recommendations for MAP.

CAR monitoring and determining MAPopt, LLA, and ULA depend on spontaneous fluctuations in blood pressure and sufficient span of blood pressure to acquire the full range of values of the functional CAR. Thus, the blood pressure range must spontaneously exceed both the LLA and ULA at least once intraoperatively. As this was not always the case, the complete blood pressure range with intact autoregulation could be determined in only 11 of 20 patients (55%), whereas in other patients, only one of the limits was determined. In only one patient, blood pressure fluctuations were minimal to such an extent that MAPopt could not be determined at all. Consequently, a MAPopt could be determined in 95% of the patients and could have been used as a target for an active MAPopt-guided hemodynamic management.

In those 11 patients where the complete blood pressure range of intact autoregulation could be determined, the span averaged 19 ± 6 mmHg, which is a smaller range than that in adults (32). Furthermore, the MAPopt of patients with similar weight and age differed significantly in our study, which was already demonstrated in adults (33). Additionally, as more datapoints were gathered throughout the intervention, the specific MAPopt changed slightly over time. Hence, careful continuous individualized monitoring may be advisable throughout the surgical procedure.

The intensity of blood pressure fluctuations during anesthesia distinctly influenced the time until the first MAPopt could be determined (Figure 2). In the patients with the strongest fluctuations, a MAPopt was determined after 18 ± 3 min of inducing GA, while in the group with the smallest fluctuation, 79 ± 48 min was needed. Other studies on patients from intensive care units required a few hours to determine a first MAPopt (34–36). Considering that blood pressure fluctuations are stronger during GA induction, our findings accord with those in the literature. Independent of MAP fluctuations, the first MAPopt was very similar to the MAPopt throughout the procedure as depicted in Figure 3; thus, the initial MAPopt may be a reliable first indicator.

In more stable patients, the long offset until a first MAPopt can be determined might not be disadvantageous, as their blood pressure may be within the target range of functional CAR (37) (Figures 2, 3).

In our cohort no hypocapnia (pCO_2_ < 35 mmHg) occurred. For this reason and due to the small cohort size, no correlation was made. Whether higher values of pCO_2_ have a relevant influence should be the subject of subsequent studies.

Using weight-adjusted blood pressure recommendations or cerebral oximetry with rSO_2_ alone, only a fraction of the periods with inadequate MAP (TICR) were identified (Figure 5). The determined mean MAPopt was 62 ± 12 mmHg, which was higher than the values recommended in the literature (26), and in 66% of the patients in whom LLA was determined, this was above the recommended value from the study by de Graaff et al. (Figure 6). Cerebral oximetry identified only a fraction of the potential risk and HVx thus seems more sensitive in detecting possible hypotension (Figure 5). One patient was within the range of functional CAR for most of the procedure; however, according to cerebral oximetry, the patient spent 53% of procedure time >20% below the baseline, which was considered critical. Since blood flow velocity was not determined, whether this could have been associated with a limited oxygen supply remains unclear (4). This patient was discharged without any neurologic impairment.

By contrast, CAR monitoring by NIRS-derived HVx, enables the determination of individual target blood pressure ranges according to MAPopt determination. This allows actively managing blood pressure guided by HVx during a major surgery under GA to reduce TICR where infants, toddlers. and small children are at risk of hemodynamic brain injury.

As low perioperative arterial blood pressure is associated with cerebral hypoperfusion and consecutive neurological injury, actively maintaining blood pressure in the range of intact autoregulation should theoretically contribute to preventing neurological injury; however, perioperative data are limited (27). Especially in the patients with a greater TICR, determining MAPopt was quicker and could thus be used as a quick therapy guideline (Figures 2, 3). Determining MAPopt and maintaining a functional CAR could be more relevant than weight- or age-related reference values respectively a MAP threshold based on a newborn gestational age. Time below LLA with impaired middle cerebral artery flow velocities could be reduced (38, 39).

### Limitations

First, the purely observational design and relatively small size of our cohort impair the power of the data generated.

Second, the concept of HVx as a surrogate parameter for the status of CAR remains debatable. Correlation with other indices, such as the pressure reactivity index, in clinical practice might be useful, although this parameter is obtainable only with invasive ICP monitoring (18). NIRS as a method can be limited by an insufficient slow wave power in the input signal by an interference of the skull thickness with the NIRS signal and the effects of blood volume changes on the tissue path length and locally observed sample volume.

Third, the necessity of manual artifact elimination that was performed prior to analysis also represents a major limitation for the use of NIRS as a MAP guideline. This could be overcome by pausing the measurement during manipulations on the arterial catheter, thereby making artifact elimination unnecessary. Automated artifact detection algorithms could possibly improve monitoring quality even more.

Lastly, although no significant or evident postoperative neurological deficits were identified, neither short-term detailed neurological examinations nor long-term data regarding neurological development were collected. Therefore, no correlations between MAP, distance of MAP to MAPopt, TICR, and neurological outcomes, could be determined.

Machine learning-based algorithms for automatic identification of reliable MAPopt and lower/upper limits of CA identification could improve the application during anesthesia.

To evaluate a potential beneficial effect of individual autoregulation-guided blood pressure management, a prospective, randomized trial comparing a MAPopt guided blood pressure management against standard blood pressure management is necessary.

## Conclusion

Monitoring CAR using NIRS and invasive arterial blood pressure can be used intraoperatively when performing major elective surgery in infants, toddlers, and small children under GA in order to identify individual hemodynamic targets to potentially optimize CAR. Individualization of target blood pressure ranges and patient-specific management may be necessary. Therefore, dedicated pediatric clinical trials using non-invasive CAR monitoring should be conducted.

## Data Availability

The raw data supporting the conclusions of this article will be made available by the authors, without undue reservation.
